# Bowstring Stretching and Quantitative Imaging of Single Collagen Fibrils via Atomic Force Microscopy

**DOI:** 10.1371/journal.pone.0161951

**Published:** 2016-09-06

**Authors:** Andrew S. Quigley, Samuel P. Veres, Laurent Kreplak

**Affiliations:** 1 Department of Physics and Atmospheric Science, Dalhousie University, Halifax, Canada; 2 School of Biomedical Engineering, Dalhousie University, Halifax, Canada; 3 Division of Engineering, Saint Mary’s University, Halifax, Canada; Illinois Institute of Technology, UNITED STATES

## Abstract

Collagen is the primary structural protein in animals. Serving as nanoscale biological ropes, collagen fibrils are responsible for providing strength to a variety of connective tissues such as tendon, skin, and bone. Understanding structure-function relationships in collagenous tissues requires the ability to conduct a variety of mechanical experiments on single collagen fibrils. Though significant advances have been made, certain tests are not possible using the techniques currently available. In this report we present a new atomic force microscopy (AFM) based method for tensile manipulation and subsequent nanoscale structural assessment of single collagen fibrils. While the method documented here cannot currently capture force data during loading, it offers the great advantage of allowing structural assessment after subrupture loading. To demonstrate the utility of this technique, we describe the results of 23 tensile experiments in which collagen fibrils were loaded to varying levels of strain and subsequently imaged in both the hydrated and dehydrated states. We show that following a dehydration-rehydration cycle (necessary for sample preparation), fibrils experience an increase in height and decrease in radial modulus in response to one loading-unloading cycle to strain <5%. This change is not altered by a second cycle to strain >5%. In fibril segments that ruptured during their second loading cycle, we show that the fibril structure is affected away from the rupture site in the form of discrete permanent deformations. By comparing the severity of select damage sites in both hydrated and dehydrated conditions, we demonstrate that dehydration masks damage features, leading to an underestimate of the degree of structural disruption. Overall, the method shows promise as a powerful tool for the investigation of structure-function relationships in nanoscale fibrous materials.

## Introduction

Collagen fibrils are the main load bearing element of all structural tissues in humans, and most animals. Fibrils range in diameter from approximately 50 to 500 nm, and are formed by an axially quarter-staggered packing of collagen molecules in a partially disordered, layered radial structure [1]. The molecular packing is stabilized via covalent intermolecular crosslinking [2]. The staggered axial registration of molecules gives rise to an observable banding pattern with a 67 nm repeat known as the D-band [3, 4]. Collagen molecules are triple-helical structures, in which three individual alpha chains in a polyproline II conformation coil around each other [5, 6]. Within an assembled fibril, collagen molecules are surrounded by a jacket of bound water molecules, separating collagen molecules laterally [7]. The hydrated triple-helix is stabilized by both direct and bound-water mediated interchain hydrogen bonding [8], stereoelectronic effects [9], and hydrophobic interactions [10].

Owing to their importance and pervasiveness throughout the animal kingdom, understanding collagen fibril structure-function relationships is of significant interest. The most direct way to explore these is to conduct mechanical experiments on single collagen fibrils. While directly manipulating individual collagen fibrils in their native, hydrated state is difficult due to their nanoscale size, within the last decade several techniques have been developed that can accomplish certain tensile experiments. These include: microelectromechanical systems (MEMS) based devices [11–13], vertically oriented atomic force microscopy (AFM) pulls [14–18], and optical tweezer based methods [19].

The existing techniques for manipulating single fibrils are capable of measuring the forces involved, and each has yielded important information. Optical tweezers have been used to bend fibrils, causing axial strains up to 0.5%, yielding valuable measurements of low-strain modulus [19]. However, collagen fibrils may have fracture strains greater than 60% [11, 13], and a non-linear stress-strain response [11, 13, 18]. In this regard, the vertical AFM pulling technique and the MEMS based technique are both capable of measuring the mechanics of fibril elongation all the way to rupture, as well as some viscoelastic properties [12, 14–18]. One important limitation of the vertical AFM pull is that fibrils must be ruptured before AFM imaging is possible because fibrils are glued between AFM probe and substrate [18]. Consequently, the structural effects of sub-rupture extensions cannot be studied. For the MEMS based technique, a new MEMS device must be fabricated for every fibril tested [11–13], limiting the practicality of conducting experiments with sufficient sample size.

In this report we present a new technique for stretching single collagen fibrils in tension. After fixing single fibrils at multiple points to a glass substrate, we employ an AFM to stretch the fibrils in a bowstring geometry. During stretching, fibril extension is viewed using differential interference contrast (DIC) microscopy, providing an accurate, real-time measure of fibril strain. Post-loading structural assessment of the fibrils is then conducted using AFM. While this method currently has its own limitation—an inability to capture force data during loading—it overcomes many of the limitations faced by previous techniques. Importantly, using our new technique it is possible to extend fibrils to target sub-rupture strains, and then conduct post-loading structural assessment. Because multiple fibrils can be prepared for loading on the same sample dish, multiple loading experiments can be done relatively quickly, allowing larger sample sizes. Also, because both loading and imaging are done with the AFM, cyclic loading experiments with intermittent structural assessment are also possible.

Another advantage of our bowstring technique is the ability to prepare multiple test segments along a single fibril, and then expose each segment to a different mechanical treatment prior to structural assessment. We use this approach here to demonstrate the technique’s utility. In doing so, we show that collagen fibrils from the digital extensor tendon of adult steer undergo significant structural change when nondestructively strained by just a few percent following dehydration and rehydration, and that localized sites of permanent damage nucleate along ruptured segments.

## Materials and Methods

### Tissue acquisition and collagen fibril extraction

The forelimb of a 24–36 month old steer was collected fresh from a slaughterhouse (Oulton farm, Nova Scotia, Canada), where the animal was killed for food. The forelimb was transferred on ice to the laboratory for dissection, where the common digital extensor tendon (CDET) was immediately removed and stored at −86°C. To extract individual collagen fibrils, the tendon was thawed and the epitenon removed using a razor blade. In phosphate buffered saline (PBS), the remaining core of the tendon was splayed open with fine tipped glass rods and metal tweezers, releasing individual collagen fibrils into the PBS. 1 mL aliquots of the PBS/collagen fibril mixture were pippetted into glass dishes, which were left stationary for 30 minutes to allow the suspended fibrils to sink and adhere to the glass substrate. The adhered fibrils were then triple-rinsed using deionized water and dried under compressed nitrogen. Glass dishes were then stored in a dessicator for 5–7 days.

### Glue fixation

**Fig 1:**
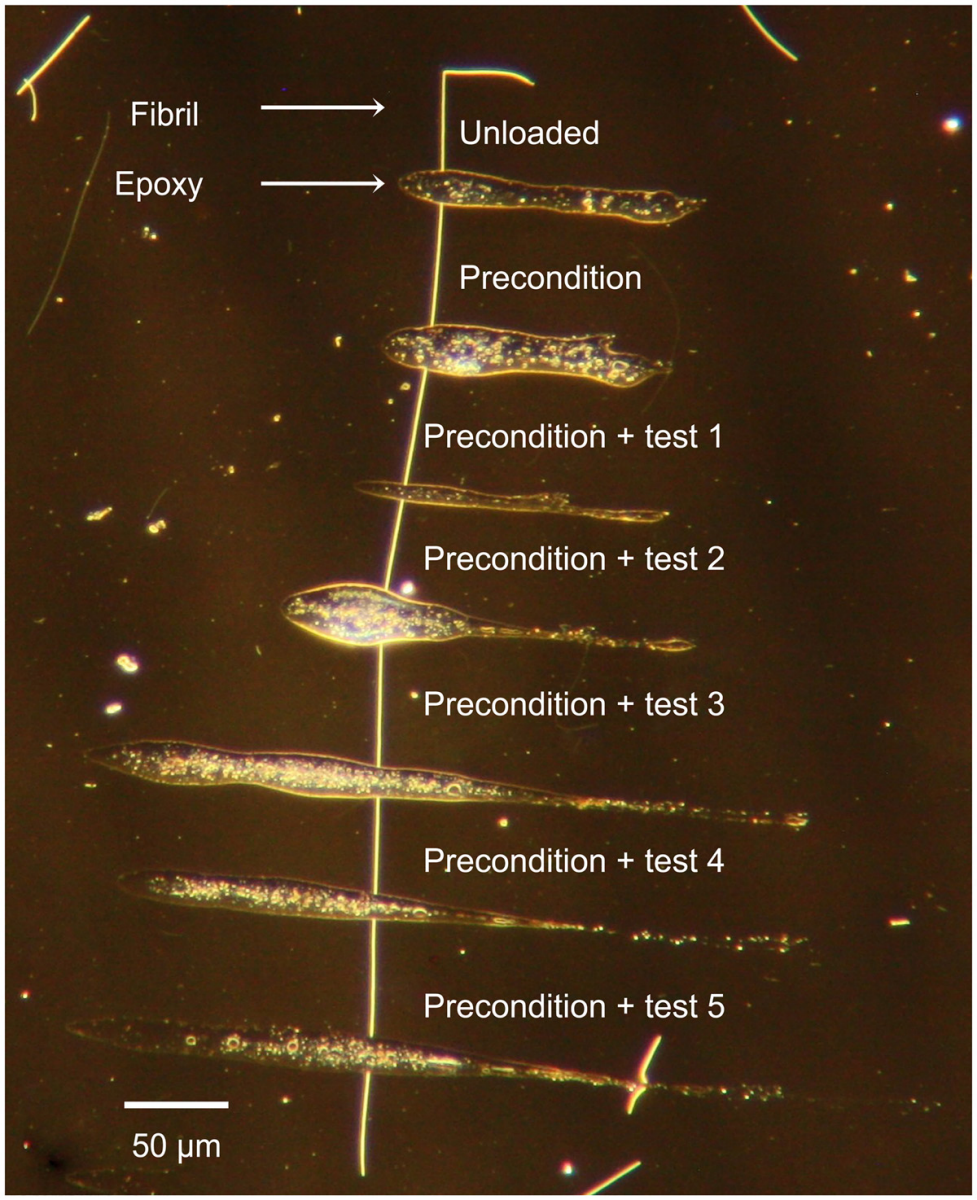
A single fibril shown prior to any tensile testing, subdivided into mechanically isolated segments using epoxy strips. Each segment underwent a unique mechanical treatment: segments were preconditioned to 3-4% strain, followed immediately by extension to a prescribed target strain ranging from 5 to 18%. One segment per fibril underwent preconditioning only, and the ends served as an unloaded comparison.

Using optical dark field microscopy, we identified straight fibrils that were ∼500 *μ*m long. Once a suitable fibril was found, a large drop of 5 minute epoxy mix (LePage, USA) was placed at the edge of the sample dish. A custom made, fine tip glass rod controlled using a micromanipulator was dipped into the epoxy, gathering some on the tip. The glass tip was pressed onto the surface twice to remove excess epoxy before depositing strips across the fibril. Regularly spaced epoxy strips were applied along each selected fibril, creating a series of 50 *μ*m long, mechanically isolated segments per fibril (Fig 1). The 50–100 *μ*m long ends of each fibril were left alone to serve as an unloaded comparison. The sample dish with glued fibrils was placed in a desiccator for 12 hours. For this report we used four different fibrils spread over two sample dishes.

### Tensile loading

**Fig 2:**
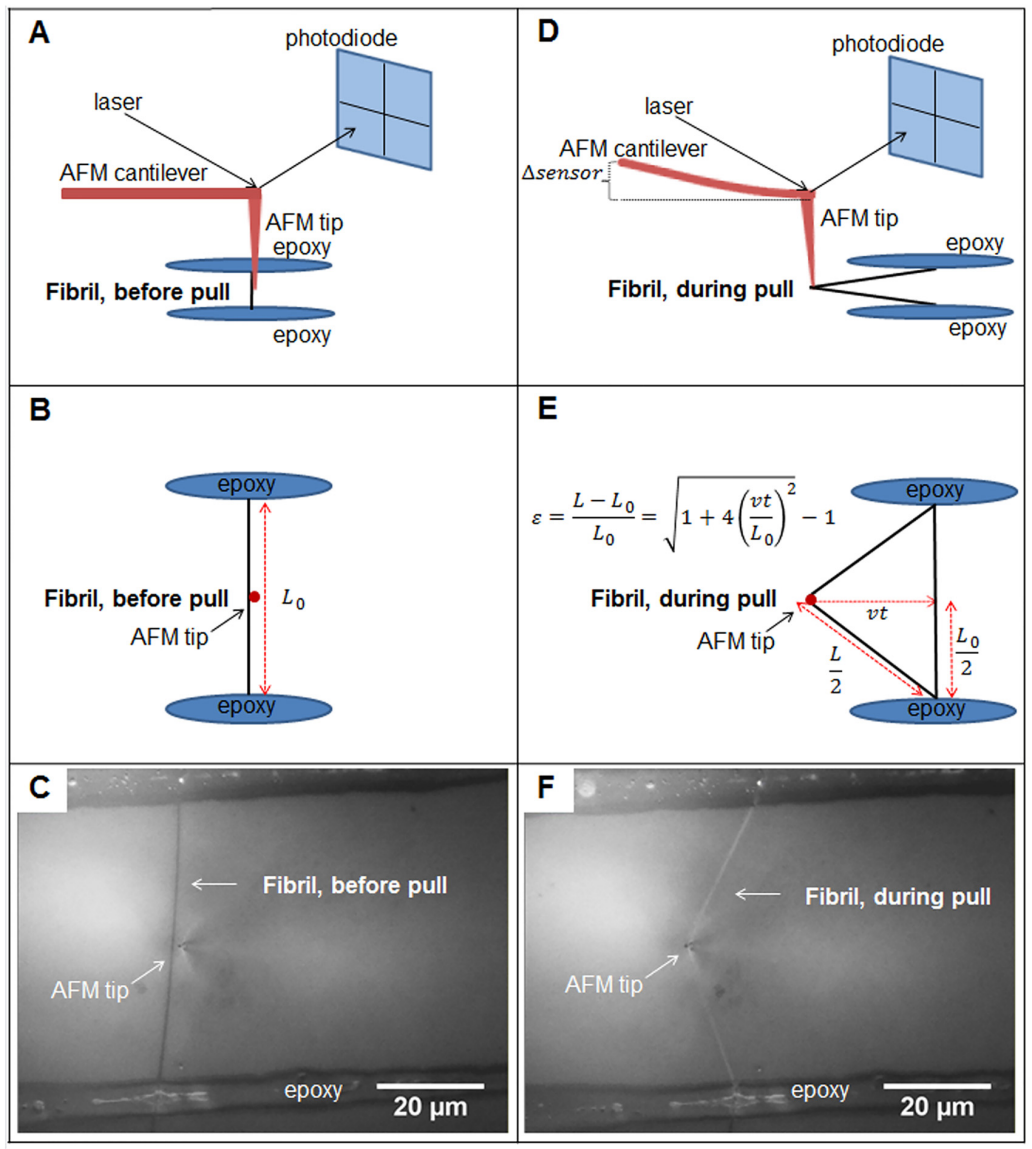
An isolated collagen fibril segment before (A-C) and during (D-F) a tensile mechanical test. To perform the test, the AFM tip remained stationary and the underlying stage was moved horizontally at a constant velocity of 1 *μ*m/s. Panels A and D show how the position of the laser on the photodiode was used as the feedback parameter to control the vertical force applied by the AFM tip. Panel E shows the geometric approximation of the fibril strain as a function of elongation time, stage speed, and segment length. Panels C and F are DIC microscopy images of a real fibril segment. Each isolated fibril segment and its two glue strips were visualized before manipulation, which allowed the midpoint position of each fibril segment to be determined using a ruler on the screen. After engaging the surface, the position of the AFM tip was realized (C), and was adjusted to match the position of the measured midpoint.

Sample dishes were removed from the dessicator, filled with 3 mL of PBS, and placed on the stage of a Catalyst AFM (Bruker, USA) mounted on an IX71 inverted microscope (Olympus, USA). The fibrils were then left to rehydrate for one hour prior to any physical manipulation. After one hour, the cantilever axis (Bruker TAP525A, nominal spring constant 200 N/m) was aligned perpendicular to the fibril axis to avoid any interference with the glue lines. The mechanical testing procedure was captured optically on a CCD camera at a rate of 1 frame/second using DIC microscopy and a 100X, 1.3 NA objective (Olympus, USA). For each segment, the AFM tip was centered between epoxy strips optically, and a downward force of ∼100 *μ*N was applied at the tip location (Fig 2) to keep the tip in contact with the substrate. The AFM stage was moved at a constant speed of 1 *μ*m/s for both loading and unloading, resulting in a fibril strain rate of ∼ 1%/s.

The mechanically isolated segments within individual fibrils were stretched to different target strains. One segment received a preconditioning cycle only, where the segment was stretched to 3-4% strain and unloaded at the same controlled rate. The remaining segments were preconditioned in the same way, and then immediately stretched to target strains ranging from 5 to 18%, from which they were unloaded at the same controlled rate (S1 Movie in the supporting material.) The determination of maximum fibril strain for each pull was carried out using frames from the recorded video and ImageJ software (version 1.45s, National Institutes of Health, USA). The error associated with maximum strain is dominated by the uncertainty in pull time, which was limited by our camera frame rate of 1 frame per second. The horizontal error bars on maximum strain are determined by standard error propagation on the formula for strain provided in Fig 2E. In total, 23 tensile tests were attempted across four fibrils.

### Hydrated AFM imaging and nanomechanical analysis

**Fig 3:**
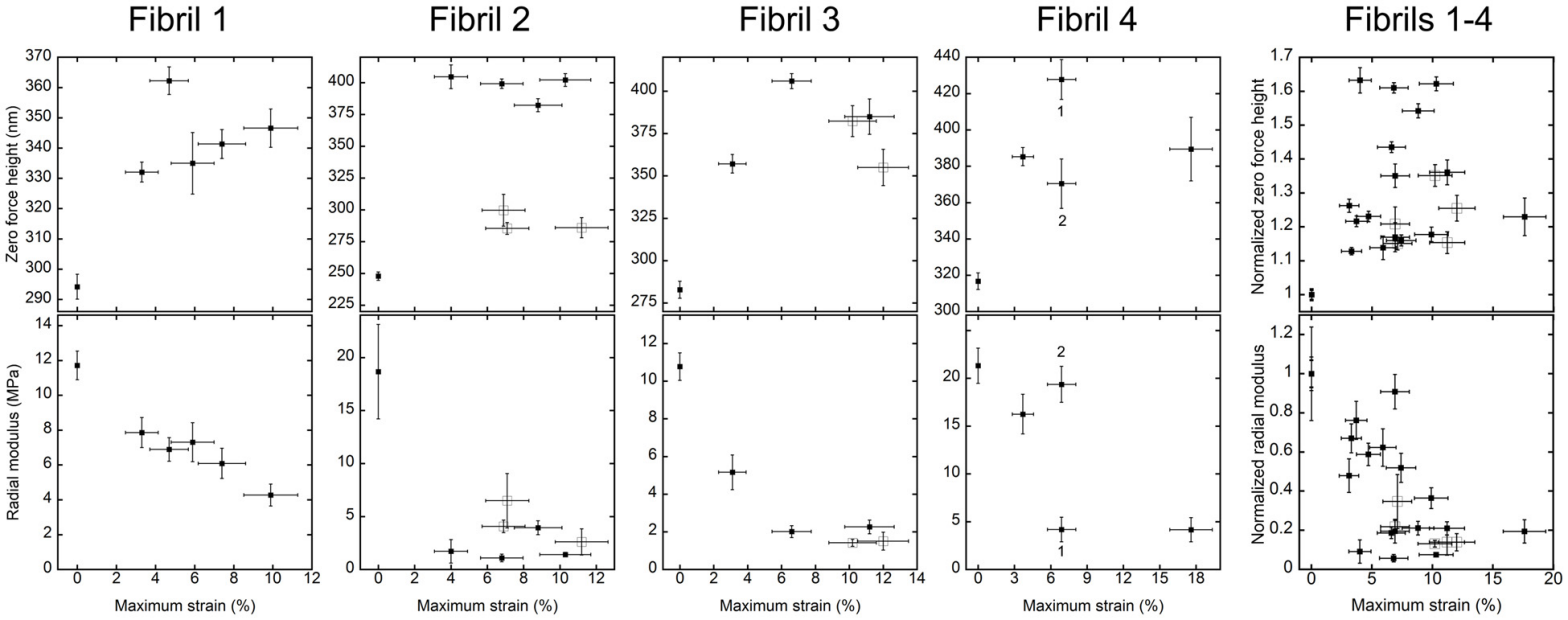
Zero force height and radial modulus vs. maximum strain achieved during tensile testing for each fibril segment (data shown as mean ± SD, n = 256 per segment). The horizontal error bars were derived from the video time resolution. The data points at 0% strain are the unloaded comparison segments from each respective fibril, and the first data points above 0% strain are the segments that underwent preconditioning only. Filled symbols represent segments that achieved their respective target strains without rupture. Open symbols represent fibril segments that ruptured. For one segment on Fibril 4, two QNM images from different segment locations (denoted by 1 and 2) yielded very different height and modulus measurements, and are therefore included separately. The plots in the rightmost column show the combined data for all four fibrils, with the height and radial modulus of each segment normalized to the unloaded values for that fibril.

After mechanical testing and while still in the PBS bath, each fibril was left for two hours on the AFM stage to re-adhere to the glass substrate. Using a Bruker ScanAsyst fluid+ probe (spring constant 0.7 N/m), force-distance curves were gathered for each image pixel using Bruker’s PeakForce Quantitative Nanomechanical Mapping (QNM) mode with an indentation speed of 1200 *μ*m/s, a 0.5 Hz raster scan frequency, and a constant peak force between 5 and 12 nN. The true height of the fibril apex was estimated by adding the QNM mode height and deformation images, and is referred to as the zero force height. The retract part of the force-distance curves along the apex of the fibril segments were fitted using the Sneddon indentation model and a Poisson’s ratio of 0.5, in order to extract a radial modulus value for each fibril segment. In order to minimize effects of the glass substrate, the force curves were fit up to 10% indentation of the zero force height [20]. Force-distance curve analysis was performed using SPIP software (version 6.3.0, Imagemet, Denmark). The vertical error bars on zero force height and radial modulus in Fig 3 are the standard deviations of 256 individual measurements made along a 5 *μ*m piece of each segment.

### Dehydrated AFM imaging

For greater structural detail, fibril segments were imaged in the dehydrated condition after hydrated AFM imaging was completed. The PBS was removed from each sample dish, which was then rinsed three times with deionized water and dried under compressed nitrogen. A ScanAsyst fluid+ probe was then used in QNM mode to image each segment in the dehydrated state at a scanning frequency of 0.125 Hz.

## Results

### Overview of experimental success

In total, we tested 23 mechanically isolated segments from four collagen fibrils. During extension, five segments ruptured. The ruptures occured at strains ranging from 6.9 to 12.0% (mean ± SD of 9.5 ± 2.4%, n = 5). For three segments, the AFM tip hopped over the fibril. These eight segments (five ruptures, three hops) were included in our analysis; the maximum strain reported corresponds to the instant before the rupture or hop, respectively. Two other segments were not included due to imaging difficulties, and one segment was discarded because the AFM tip hopped over it more than once. Overall, both mechanical testing and imaging were successfully completed on 20 of the 23 attempted segments.

### Nanomechanics of hydrated fibrils after stretching

Zero force height and radial indentation modulus were measured by hydrated AFM imaging in PBS after tensile testing. Measurements made on the unmanipulated ends of each fibril served as unloaded controls. Values of zero force height and radial indentation modulus for the unloaded controls and mechanically tested segments are shown in Fig 3 as a function of the maximum strain achieved. For the unloaded fibril segments, both the average value and the standard deviation of the radial modulus measured over 5 *μ*m of fibril length) in this study agree with the previously published radial modulus measurements on hydrated rat and bovine tail tendon fibrils [20, 21].

**Fig 4:**
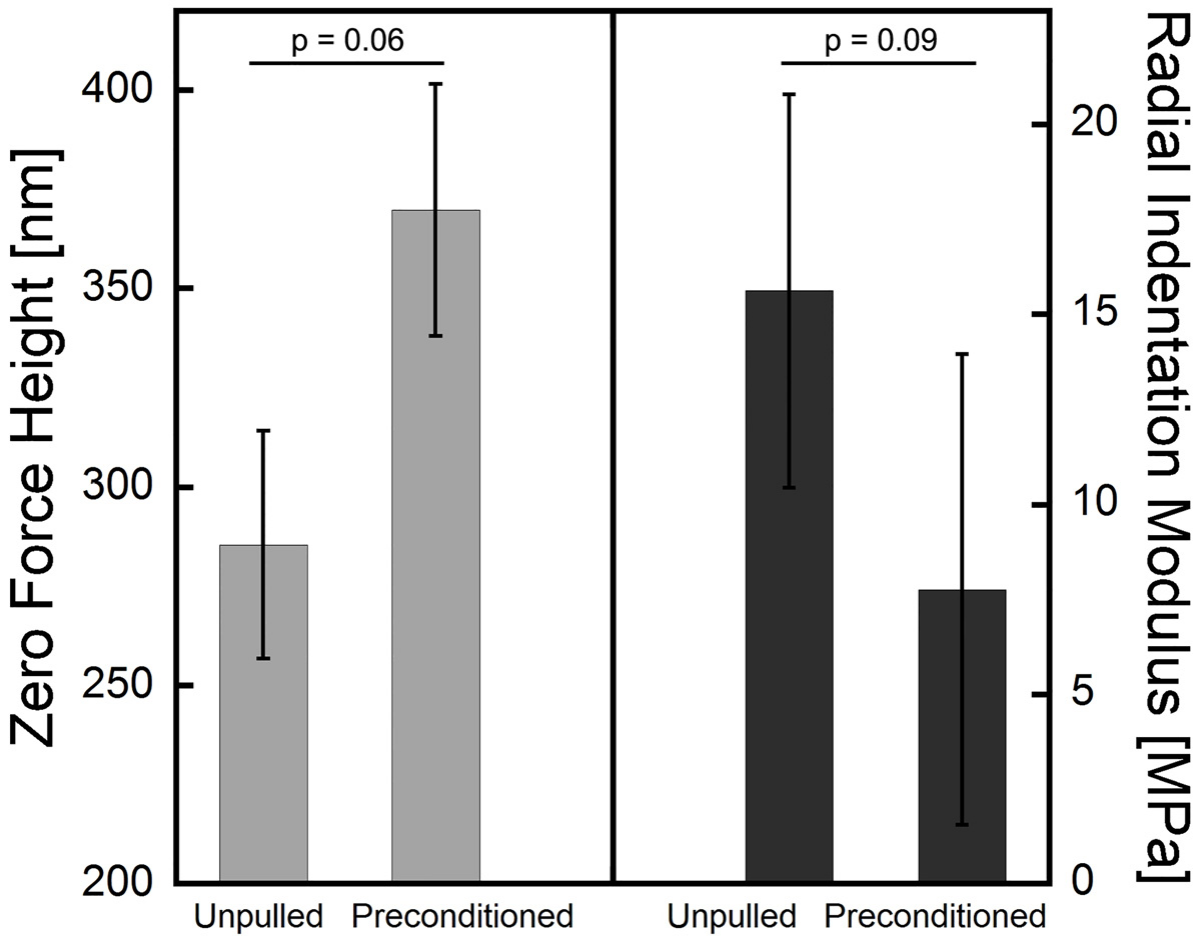
Concurrent change in average zero force height and radial indentation modulus in response to the preconditioning cycle (one cycle of 3-4% strain) for the four fibrils tested (mean ± SD, n = 4). The p-values shown were calculated using a two-tailed, matched-pairs t-test (JMP software, version 12.0.1, SAS Institute Inc.).

Fibril segments that underwent only preconditioning experienced two important changes in comparison to the unloaded control segments: on average, preconditioned segments increased in zero force height by 30% and their radial modulus dropped by 50% (Fig 4).

After undergoing preconditioning, 16 segments were successfully stretched to strains ranging from 5 to 18%. Importantly, unruptured segments returned to their original, unstretched lengths. After increasing in height in response to preconditioning, the height of unruptured fibril segments did not consistently increase further with increasing maximum strain. The height increase that occured between the unloaded control and preconditioned segments was larger than the height increase between preconditioned segments and those extended to the maximum strain without rupture. Within this overall relationship, the trend of the zero force height varied: for Fibril 1, a steady increase occured above the preconditioning strain. For Fibril 2, the zero force height plateaued sharply after preconditioning. Fibrils 3 and 4 both demonstrated height increases at intermediate strains above the preconditioning level, followed by a relative decrease in zero force height for the maximally strained segment. For each fibril, the trend of zero force height was mirrored by a reciprocal trend in the radial indentation modulus. (Fig 3). The modulus is a measure of material relaxation; a decrease in modulus is expected for a swollen fibril [20].

The ruptured segments behaved differently than their similarly strained, unruptured counterparts. While the five ruptured segments had similar radial modulus measurements to unruptured segments, they generally demonstrated a lower zero force height compared to unruptured segments (Fig 3, Fibrils 2 and 3).

### Morphology of fibrils after stretching

**Fig 5:**
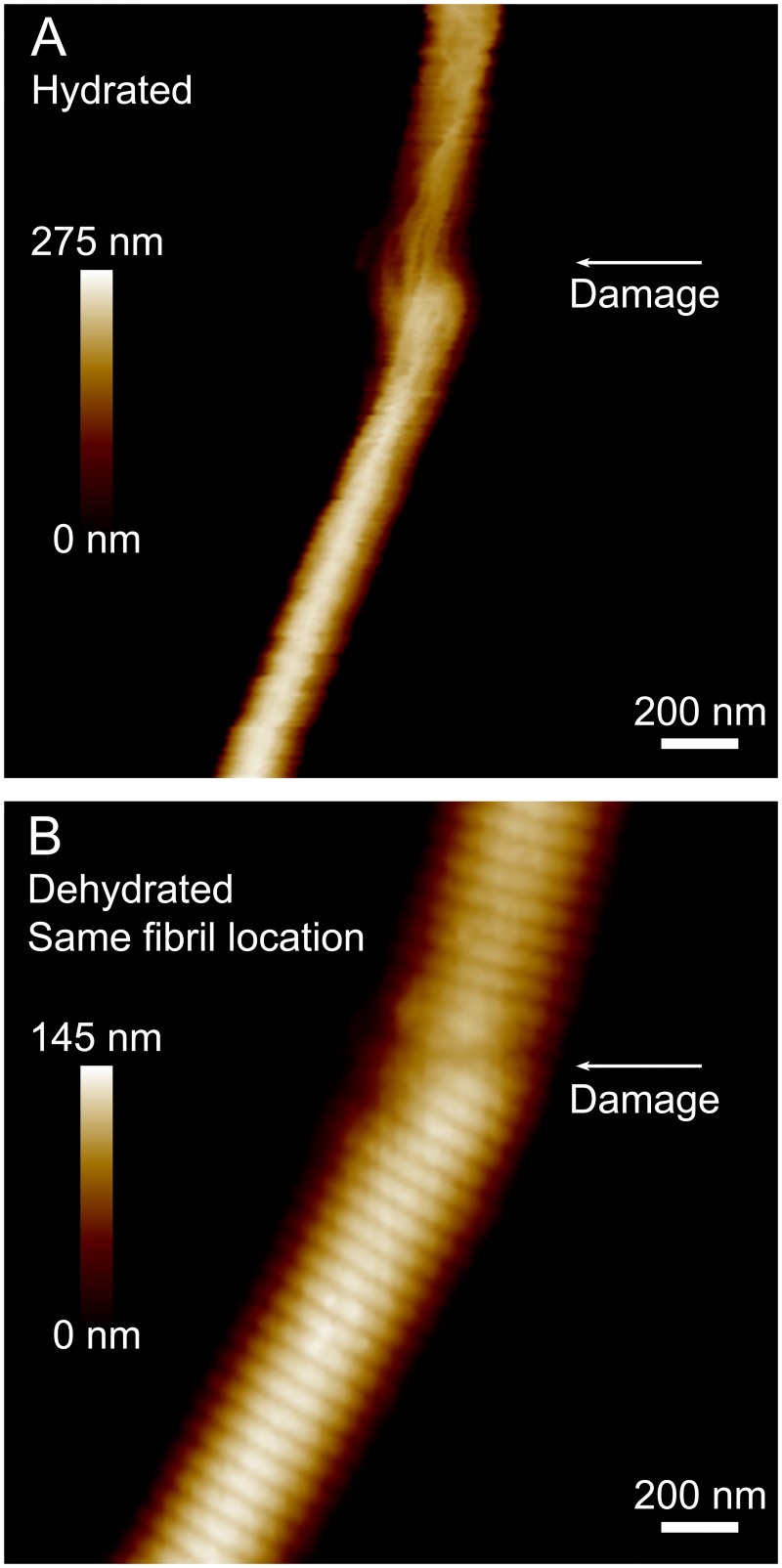
Comparative height images for a ruptured fibril segment, imaged away from the broken end, measured hydrated in PBS (A) and then dehydrated (B). In this case, dehydration reduced the fibril cross sectional area by a factor of 1.3. A singular, localized event of permanent damage was observed in both states. On dehydration, the D-band appeared everywhere along the length of the fibril except at locations of damage (arrow), where significant disruption to molecular packing had evidently occurred.

Both ruptured and unruptured fibril segments were morphologically different when imaged hydrated vs. dehydrated. D-banding was typically not visible for hydrated fibrils, but became readily apparent after dehydration, with the fibrils appearing flatter and wider (Fig 5A vs. 5B). In terms of permanent damage, ruptured and unruptured segments were distinct from one another: discrete zones of deformation were observed exclusively and ubiquitously on ruptured segments. The damage on ruptured fibrils appeared more pronounced during hydrated imaging than during subsequent dehydrated imaging. In the hydrated state, dissociated subfibrillar elements are clearly visible in the fibril shown in Fig 5A, but are no are no longer visible in the dehydrated state. Still visible, though, is a fault line that runs across the fibril, straddling three continuous D-band periods (Fig 5B). The longitudinal extent of this damage sites was larger in the hydrated state, and highlights how only air drying may lead to an underestimate of the damage incurred by a ruptured fibril.

**Fig 6:**
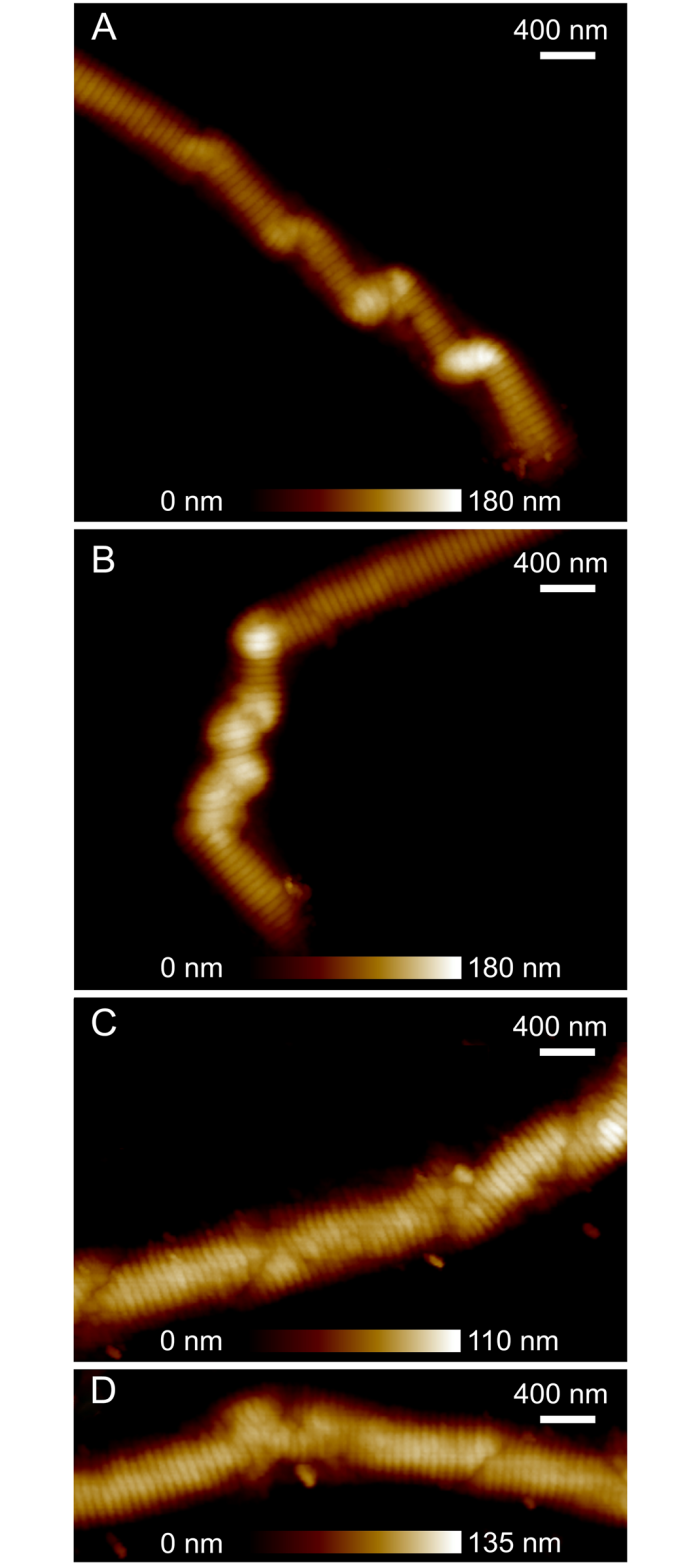
Two distinct forms of permanent damage occured in ruptured fibril segments. Near ruptured ends, the damage appeared to have a helical nature (Panels A and B), which was not present on damage sites further away from ruptured ends (Panels C and D). Panels A and B are from separate ruptured segments from Fibril 3, and Panels C and D each depict one half of a ruptured segment from Fibril 2, which ruptured in its middle near the AFM tip.

Instances of permanent damage that occurred less than 5 *μ*m away from the broken ends of a fibril segment were often morphologically distinct from those that occurred more than 5 *μ*m away. Close to the rupture site, multiple periodic bulges in height were seen at precisely the same locations as changes in fibril trajectory, suggesting that each of these localized deformations involved a helical twisting of the fibril (Fig 6A and 6B). The broken ends appeared frayed, flatter, and wider than the rest of the fibril. Further away from the rupture site, discrete zones of deformation were sometimes visible, but were not characterized by localized bulges in fibril height or direction changes, suggesting that the fibrils did not helically turn over themselves at these damage locations (Fig 6C and 6D).

## Discussion

In this study, we showed that an AFM can be used to load, unload, and subsequently image collagen fibrils, with both non-destructive and destructive capabilities. By using DIC imaging in tandem with the AFM, manipulations were monitored in real time, giving immediate access to rupture location and strain. We successfully loaded and imaged 20 out of 23 mechanically isolated fibril segments. The combination of high success rate, low experimental time required per test, ability to image after non-destructive loading, and real-time visualization makes this unique methodology an excellent tool for studying nanoscale structure-function relationships in collagen, and potentially other fibrous materials.

An obvious limitation of the method in its current form is the inability to capture usable force data during fibril extension. However, by changing the geometry of loading such that the AFM cantilever is aligned parallel to the fibril axis rather than perpendicular to it, force measurements are possible [22–26]. The torsional stiffness of the cantilever is much higher than its normal bending stiffness; therefore force sensitivity is much higher with a parallel relationship between cantilever and fibril axes. Preliminary experiments in our laboratory support this. Another minor limitation of the method is the change in instantaneous strain rate during specimen loading at constant stage velocity due to the bowstring geometry. In this report we used an unstretched segment length of 50 *μ*m and a constant stage speed of 1 *μ*m/s. Fibrils experienced an increasing instantaneous strain rate with increased extension. This effect, though, is relatively small, with fibrils experiencing an instantaneous strain rate of 1.2%/s at 5% strain, and 2.0%/s at 15% strain.

The radial indentation modulus of unloaded fibrils reported here is larger than values reported previously for hydrated fibrils [27]. The cause of this discrepancy is that our indentation rate was three orders of magnitude larger than used in that study. We have previously elucidated the dependence of radial indentation modulus on indendation rate [20], and the modulus measurements in both the current work and those from the work of Grant *et al.* [27] match the predictions of our previous work. A secondary contributing factor may be that Grant *et al.* [27] used reconstituted bovine Achilles fibrils, while our fibrils were not reconstituted.

After undergoing dehydration and subsequent rehydration, our experiments show that fibril segments increase in height and decrease in radial modulus after experiencing a small amount of tensile strain. For the unruptured fibril segments, a return to original unstretched length combined with an increase in height suggests that fibril volume may be increasing. An increase in fibril volume could be explained by the uptake of surrounding water molecules into the collagen structure, contributing to the hydrogen bonding network that stabilizes collagen molecules. Given the scale of our measurements, we cannot conclude if the additional water molecules exist within collagen molecules, between collagen molecules, or both. In computational modelling of an 8 nm segment of the collagen mimmicking molecule [(*Gly* − *Pro* − *Hyp*)_10_]_3_ pulled in water, intrahelical hydrogen bonds are shown to rupture above 10% strain, and are replaced by around 5 times more protein-solvent hydrogen bonds [28]. Native intrahelical hydrogen bonds being broken and replaced by a higher number of protein-solvent hydrogen bonds is one explanation for the increase in fibril height we observed. It is also possible that the network of amino acid side chains and sugar chains that extend from the central helix of collagen molecules prevent full rehydration after the dehydration step needed for glue application. If the side chains of neighboring molecules become tangled or weakly bonded upon dehydration, the recovery of the native water jacket may be hindered until strain induced molecular agitation untangles and separates the chains during preconditioning.

The damage sites observed in this study have both similarities and differences in comparison to a mode of fibril damage called discrete plasticity that occurs in tendons in response to rupture [29], and progresses in disruption intensity with repeated subrupture overloading [30]. Morphologically, discrete plasticity presents as a longitudinal series of quasi-periodic kinks, in which collagen molecules are denatured [31]. Comparing the fibril damage in the current study to discrete plasticity induced by whole tendon rupture, both consist of multiple sites of localized damage, which can appear as kinks that have a helical twist. In the current study, the subfibrillar components that are apparent in the hydrated state (Fig 5) are similar to the subfibrillar components that remain at the kink sites after enzymatic digestion of denatured collagen [29]. However, the damage caused by fibril rupture in the current study did not extend along the full length of the 50-*μ*m-long fibril segments, whereas discrete plasticity damage typically extends for hundreds of microns along individual fibrils [29]. This difference may be due to stress localizations at the glue fixation points or AFM tip causing the fibrils ruptured in this report to break prematurely. The rupture strain measured in our experiments is low compared to published values [18]. In that same study, single fibrils ruptured at 100%/s were either completely disrupted (evidenced by loss of D-band and delamination of an outer layer) or were largely intact [18]. The damage on completely disrupted fibrils in that study is both longitudinally extensive, and presents with surface damage, consistent with discrete plasticity. It is also possible that different single fibril loading methodologies (including preconditioning, geometry, and strain rate) produce different stress localizations, resulting in distinct structural alterations.

The current study suggests that the type of damage depends on proximity to rupture location. Due to a tension-torsion coupling arising from the helical ordering of the microfibrils and subfibrils that make up collagen fibrils [32–35], it is likely that axial stress may have an associated torsional component. The stored torsional energy would induce fibril rotation about the longitudinal axis after rupture, and the fibril’s ability to freely rotate locally should depend on promixity to a free end. Thus the notion of inducing a torsional energy via axial strain is consistent with the damage that we observe: in close proximity to the ruptured ends, the fibrils appear to undergo helical kinking. In contrast, damage sites away from the ruptured ends do not have a twisted morphology. Upon rupture, the fibril’s broken ends would be least constrained, therefore having greater ability to rotate and release the torsional energy induced by the axial strain.

## Conclusion

We have developed an AFM-based methodology for loading and subsequently imaging collagen fibrils, with both rupture and subrupture capabilties. Considering the high success rate of our technique, it will be useful for studying structural-mechanical relationships in a wide range of nanoscale fibres. In the case of collagen fibrils, this method will enable the direct assessment of how factors such as fibril diameter, crosslinking, and pathology affect fibril function.

## Supporting Information

S1 Movie Recorded video of a tensile experiment on a single collagen fibril. (AVI)
